# Risk of post-vasectomy infections in 133,044 vasectomies from four international vasectomy practices

**DOI:** 10.1590/S1677-5538.IBJU.2023.0143

**Published:** 2023-05-20

**Authors:** Samuel Lawton, Alison Hoover, Gareth James, Simon Snook, Diana Soraya Torres Quiroz, Michel Labrecque

**Affiliations:** 1 Emory University Rollins School of Public Health Atlanta United States Emory University Rollins School of Public Health, Atlanta, United States;; 2 Emory University School of Medicine Atlanta United States Emory University School of Medicine, Atlanta, United States; 3 Primary Care United Kingdom Association of Surgeons in Primary Care, United Kingdom, UK; 4 SNIP Vasectomy Clinics New Zealand SNIP Vasectomy Clinics, New Zealand; 5 Profamilia Bogota Colombia Profamilia, Bogota, Colombia; 6 Laval University Department of Family and Emergency Medicine Quebec City Canada Department of Family and Emergency Medicine, Laval University, Quebec City, Canada

**Keywords:** Vasectomy, Infections, Medical Audit

## Abstract

**Objectives:**

To estimate the risk of post-vasectomy infections in various settings and across various surgical techniques and sanitization practices.

**Patients and Methods:**

Retrospective review of the records of 133,044 vasectomized patients from four large practices/network of practices using the no-scalpel vasectomy (NSV) technique in Canada (2011-2021), Colombia (2015-2020), New Zealand (2018-2021), and the United Kingdom (2006-2019). We defined infection as any mention in medical records of any antibiotics prescribed for a genital or urinary condition following vasectomy.

**Results:**

Post-vasectomy infection risks were 0.8% (219 infections/26,809 procedures), 2.1% (390/18,490), 1.0% (100/10,506), and 1.3% (1,007/77,239) in Canada, Colombia, New Zealand, and the UK, respectively. Audit period comparison suggests a limited effect on the risk of infection of excising a short vas segment, applying topical antibiotic on scrotal opening, wearing a surgical mask in Canada, type of skin disinfectant, and use of non-sterile gloves in New Zealand. Risk of infection was lower in Colombia when mucosal cautery and fascial interposition [FI] were used for vas occlusion compared to ligation, excision, and FI (0.9% vs. 2.1%, p<0.00001). Low level of infection certainty in 56% to 60% of patients who received antibiotics indicates that the true risk might be overestimated. Lack of information in medical records and patients not consulting their vasectomy providers might have led to underestimation of the risk.

**Conclusion:**

Risk of infection after vasectomy is low, about 1%, among international high-volume vasectomy practices performing NSV and various occlusion techniques. Apart from vasectomy occlusion technique, no other factor modified the risk of post-vasectomy infection.

## INTRODUCTION

Vasectomy is a minor surgery with low risk of infectious complications, especially when providers utilize the no-scalpel vasectomy (NSV) technique (1, 2). In 2012, the committee members of American Urological Association (AUA) guideline on vasectomy reviewed studies with a sample size greater than 500 patients and ultimately estimated a risk of post-vasectomy infection between 1% and 2% (3). However, the AUA review did not provide a clear definition of post-vasectomy infection, nor did it describe the types of infection that occurred. Additionally, the review did not expand on the variation of the risk of infection in relation to surgical technique and other salient clinical factors.

On June 12, 2021, World Vasectomy Day, an international non-governmental organization supporting male participation in family planning, and the Vasectomy Network, a Google Group of over 600 health professionals, organized a webinar on post-vasectomy infections to address these gaps in clinical research. During the meeting, four of the authors (ML, DT, SS, and GJ) presented the results of audits of post-vasectomy infections conducted in their large vasectomy practices and networks of practices from Canada, Colombia, New Zealand, and the United Kingdom (UK) respectively.

The objectives were to estimate the risk of post-vasectomy infection in these various settings and to assess the risk of infection according to changes in surgical techniques and sanitization practices, level of certainty of infection, vasectomy surgeons, and types of infection. Their findings are presented here.

## PATIENTS AND METHODS

### Settings

We conducted a retrospective review of the vasectomy records of patients from the four participating practices and networks of practices (henceforth referred to as practices). Table-1 describes the characteristics of the four sites. All where high-volume practices with over 3,000 vasectomies performed each year. All providers utilized the NSV technique or variants of NSV to expose the vas deferens (1, 4, 5), but differed in their occlusion techniques. With the exception of the Colombia site where patients attended a visit with a general practitioner one-week post procedure, no routine post-op follow-up was required of vasectomy patients in the other three countries.

**Table 1:** Characteristics of the four vasectomy practices/network of practices.

Country	Setting	Surgical technique	Post vasectomy follow-up	Infection prevention procedures
Canada	About 3,000 vasectomies performed yearly by three family physicians and one urologist in two primary care clinics in Quebec City area.	Local anesthesia with mini-needle (100%), vas delivery by NSV, (1) vas occluded by thermal mucosal cautery and fascial interposition with metal clip over abdominal end, testicular end left open.	Advised to contact by phone or e-mail if a problem. Office consultation if judged necessary (about 2/3 dealt by phone).	No prophylactic antibiotics, except topical antibiotics during a single year (see Table-2); Self shaving at home; Skin cleaning with sterile water 8 parts, chlorhexidine 4% one part, and alcohol 70%/99% one part; Hand washing before vasectomy session (20-25 patients) and alcohol gel used between each patient; Surgery with sterile gloves; Surgical mask not used until May 2020, implemented thereafter in response to COVID-19 pandemic;
Colombia	About 4,000 vasectomies performed yearly by four urologists in one family planning clinic in Bogota.	Local (96%) and general (4%) anesthesia, (12) vas delivery by modified no- scalpel vasectomy, (12) vas occlusion by thermal mucosal cautery and fascial interposition with silk ligature over abdominal end, testicular end left open (12, 13).	Routine visit with general practitioner one-week post- procedure.	No prophylactic antibiotics; Minimal shaving by the nurse on the day of the surgery; Skin cleaning with Chlorhexidine 4% or Povidone iodine 10%; Hand washing every 5 patients before 2016, before each patient thereafter; Surgery with sterile gloves and surgical mask.
New Zealand	About 3,000 vasectomies performed yearly by five family physicians in 13 clinics across the country.	Local anesthesia (100%), vas delivery by electrocautery no scalpel technique, (5) vas occlusion by extended electrocautery without division (Marie Stopes technique) (5).	Advised to contact by phone or e-mail if a problem. Office consultation if judged necessary (99% dealt by phone).	No prophylactic antibiotics; No shaving; Skin cleaning with Povidone iodine 8% or Chlorhexidine 4%; Hand washing before vasectomy session (20-25 patients). Alcohol gel used between each patient; Surgery with or without sterile gloves (surgeon choice); Surgery without surgical mask;
United Kingdom	About 6,000 vasectomies performed yearly by 22 to 44 general practitioners within the network of ASPC members across the country.	Local anesthesia (100%), vas delivery variable: most with modified NSV techniques, vas occlusion variable: most with extended electrocautery, with division of the vas, with or without excision (6).	No routine visits, but some do routine follow-up telephone calls and a four- month post-op questionnaire sent to each patient.	No prophylactic antibiotics; Shaving variable: about one-third no shaving, one-third trim only, and one- third shaving; Skin cleaning variable: Povidone iodine or Chlorhexidine; Hand washing before each patient; Surgery with sterile gloves; Surgery with surgical mask variable. Mostly not used until April 2020, implemented thereafter in response to COVID-19 pandemic.

NSV = No scalpel vasectomy; ASPC = Association of Surgeons in Primary Care

Infection prevention procedures used in the four settings are also described in Table-1. No patients included in the study received prophylactic antibiotics. Solutions of chlorhexidine or povidone iodine were used to sterilize the skin in all four practices. Clinical protocols for shaving, handwashing, use of sterile gloves, and use of surgical masks varied across time and settings in all four practices.

### Data sources and collection

For our analysis, we defined infection as any mention in patient medical records of any antibiotics prescribed for treating a genital or urinary condition following the patient's vasectomy. We excluded the application of topical antibiotics for minor skin infections located at the scrotal opening site. Data sources and collection processes varied across settings. Data collection periods covered 10 years in Canada, 6 years in Colombia, 3 years in New Zealand, and 13 years in the UK (Table-2).

**Table 2:** Risk of post-vasectomy infections in the four vasectomy practices/network of practices according to changes in clinical practice.

Country and audit period		Number of vasectomies	Number of infections	Changes in clinical procedures*
		n	n (%)	
**Canada**				
	Jan 2011-Nov 2015	10,590	100 (0.9) †	Excision‡, no topical antibiotics, no surgical mask
	Nov 2015-Nov 2016	2,606	20 (0.8)	Excision, topical antibiotics, no surgical mask
	Nov 2016-Nov 2017	2,365	15 (0.6)	Excision, no topical antibiotics, no surgical mask
	Nov 2017-Mar 2020	7,002	60 (0.9)	No excision, no topical antibiotics, no surgical mask
	May 2020-May 2021	4,246	24 (0.6)	No excision, no topical antibiotics, surgical mask
**Total**		**26,809**	**219 (0.8)**	
**Colombia**				
	Jan 2015-Dec 2015	3,347/2,311 §	105 (3.1/4.5) †	LE + FI, hand washing for every 5 patients
	Jan 2016-Dec 2018	9,584/7,371	233 (2.4/3.2)	LE + FI, hand washing before each patient
	Jan 2019-Dec 2020	5,559/4,355	52 (0.9/1.2)	Cautery + FI, hand washing before each patient
**Total**		**18,490/14,037**	**390 (2.1/2.8)**	
**New Zealand**							
	Apr 2018 – Feb 2019	1,448	7 (0.5) †	Skin cleaning Povidone iodine, surgery with or without sterile gloves
	Apr 2018 - Mar 2021	9,058	93 (1.0)	Skin cleaning Chlorhexidine, surgery with or without sterile gloves
**Total**		**10,506**	**100 (1.0)**	
	Apr 2018 - Mar 2021	9,590	88 (1.3) †	Skin cleaning Chlorhexidine or Povidone iodine, surgery without sterile gloves
	Oct 2020 - Mar 2021	916	12 (0.9)	Skin cleaning Chlorhexidine or Povidone iodine, surgery with sterile gloves
**Total**		**10,506**	**100 (1.0)**	
**United Kingdom**				
	Jul 2006-Jun 2008	7,463	129 (1.7) †
	Jul 2008-Dec 2008	2,406	42 (1.7)	
	Jan 2009-Dec 2009	3,411	35(1.0)	
	Jan 2010-Dec 2010	6,116	82 (1.3)	
	Jan 2011-Dec 2011	5,583	66 (1.2)	
	Jan 2012-Mar 2013	7,363	99 (1.3)	
	Apr 2013-Mar 2014	6,774	92 (1.4)	
	Apr 2014-Mar 2015	6,581	95 (1.4)	
	Apr 2015-Mar 2016	6,327	66 (1.0)	
	Apr 2016-Mar 2017	7,832	114 (1.5)	
	Apr 2017-Mar 2018	7,779	91 (1.2)	
	Apr 2018-Mar 2019	9,604	96 (1.0)	
**Total**		**77,239**	**1,007 (1.3)**	

LE +FI = vas occlusion by ligation/excision combined with fascial interposition on testicular segment (12); Cautery + FI = vas occlusion by thermal mucosal cautery combined with fascial interposition on abdominal segment (13).

* Empty lines indicate that there were no changes during audit periods or highly variable (UK). Refer to Table 1 for procedures routinely used in each country during audit periods.

† Chi square tests: Canada, 4 df = 6.6 p= 0.16; Colombia, 2 df = 59.0, p < 0.00001, 2015-18 *vs*. 2019-20 1 df = 53.0, p<0.00001; New Zealand, skin cleaning 1 df =3.91 p=0.05, and type of gloves 1 df =1.37 p=0.24; United Kingdom 11 df = 30.85 p =0.001

‡ Excision a 0.5 cm segment of vas deferens from the testicular end in order to remove damaged vas tissue and obtain a clean open testicular end.

§ All patients vasectomized/patients attending routine 7-day follow-up

In the Canadian practice, attending physicians routinely documented any post-vasectomy contact with patients using an electronic medical record specifically designed for vasectomy. Author ML identified all patients with post-vasectomy contact and searched for patients with any infectious conditions recorded in the “Diagnosis of complication” field within the electronic medical record. In addition, ML performed a free-text search with following words “infection” and “antibiotic”, as well as “levaquin®”, “clavulin®”, “cipro®” as the most commonly prescribed antibiotics in his practice. Patients with minor localized skin opening infection and an infectious condition with an evidently absent link with the vasectomy procedure (e.g., acute prostatitis 397 days after vasectomy) were excluded.

Once an instance of antibiotic prescription was identified, ML documented the date of the vasectomy, the vasectomy provider, type of infection, presence of hematoma, the date an antibiotic was prescribed, the healthcare provider who authorized the prescription, and certainty of infection (high/low). Certainty of infection was assessed on the attending physician's determination (certain or probable = high; possible = low) or signs, symptoms, and timing described in the record. Patients with fever, scrotal abscess, severe or increasing moderate scrotal pain with skin oedema and erythema and urinary symptoms occurring in the first week after vasectomy were classified as having high probability of infection. ML also documented the dates of the following changes taking place in the practice over the years: topical antibiotics applied on the skin opening, excision of a short vas segment, and the use of surgical masks.

In Colombia, author DT conducted a string search for the words “ciprofloxacina”, “norfloxacina”, “cefalexina”, and “gentamicina” in the electronic medical records of patients who attended one-week post-procedure routine follow-up visits, or any other visits related to vasectomy to identify patients who received these antibiotics. This search excluded patients who received prophylactic antibiotics for large hematomas (n=22) and patients who had both circumcision and vasectomy performed on the same day (n=24). These patients represented 0.25% (46/18,536) of the vasectomies performed in Colombia. DT recorded the dates of changes in hand sanitation procedures and vasectomy occlusion techniques.

The New Zealand practice maintains a post-operative contact spreadsheet. Author SS reviewed the post-vasectomy paper-based clinical records of patients who received consultation for a post-vasectomy concern to identify those who were prescribed an antibiotic. Patients who were given antibiotics with (e.g. swelling, erythema, fever) and without (no increasing pain) strong evidence of infection were considered to have a high- and low-probability of post-vasectomy infection, respectively. SS also documented the dates of changes in the type of skin disinfectant used (4% solution of chlorhexidine or 10% of povidone iodine) in the practice, and the use of sterile or non-sterile gloves during vasectomy.

In the UK, author GJ reviewed the vasectomy audit database of the Association of Surgeons of Primary Care (ASPC). Since 2006, ASPC annually collects data from approximately 30 vasectomy providers each year (6), Surgeons send a web-based questionnaire to each patient four months after the vasectomy and report the completed questionnaire to ASPC. Risk of infection was determined via affirmative responses to a question asking if any clinician (e.g. vasectomy provider or general practitioner) prescribed an antibiotic following vasectomy.

In addition to extracting responses from this survey, GJ conducted three analyses of data available in the ASPC audit database to validate the frequency of infection. In the first analysis, surgeons participating in the audit process between 2012 and 2019 were divided into three groups reflecting the return rate of their four-month post-operative questionnaire to ascertain the homogeneity of infection risk. In the second analysis, GJ retrieved data from a single vasectomy clinic where 278 patients had a vasectomy in 2016-2017. Patients were contacted and inquired about their use of antibiotics. In the third analysis, GJ consulted data from another clinic where the vasectomy surgeon and administrative team reviewed the general practitioners’ electronic record of each patient who had a vasectomy in 2017-2018. Patients who had clinical visits with their physician or were prescribed antibiotics for any reason within four weeks of the procedure were recorded for analysis.

At each site, vasectomy providers gave authorization to access their data or personally provided data to conduct the retrospective audit. We did not seek approval of ethical review boards as it is not required for clinical audits (7–9). Apart from nominal support from the vasectomy practice organisations in each country (Vasectomie Québec in Canada, Profamilia Bogota in Colombia, SNIP Vasectomy Clinics in New Zealand, ASPC in UK) no funding or financial support was received to conduct this study.

### Data analysis

Analysis of each dataset was performed with respect to variations in clinical procedures over time (Table-2) and to different providers (Table-3). Infection risks were evaluated using a denominator of the total number of vasectomies performed during the study period at each site. In the Colombia practice, an additional risk calculation was performed using the number of patients who returned for the routine one-week follow-up visit. Differences between infection risk according to time and clinical procedures were evaluated with chi-square test or Fisher's exact test where chi-square is not applicable (10). We considered p-values less than 0.05 to be statistically significant.

**Table 3:** Risk of post-vasectomy infections in the Canada and New Zealand practices according to vasectomy surgeons and the certainty of infections.

Country and Physician		Certainty of Infections			Number of Vasectomies
		High n (%)	Low n (%)	Total n (%)	n
**Canada (2011-2021)** *					
	1	53 (0.3)	54 (0.3)	107 (0.6)	17,115
	2	25 (0.5)	26 (0.6)	51 (1.1)	4,553
	3	16 (0.4)	43 (0.9)	59(1.3)	4,537
	4	2 (0.3)	-	2 (0.3)	604
**Total**		**96 (0.4)**	**123 (0.5)**	**219 (0.8)**	**26,809**
**New Zealand** (2018-2021)†					
	1	15 (0.3)	19 (0.4)	34 (0.7)	4,575
	2	12 (0.5)	19 (0.8)	31 (1.2)	2,481
	3	10 (0.4)	13 (0.5)	23 (0.9)	2,534
	4	2 (0.2)	8 (1.0)	10 (1.2)	807
	5	1 (0.9)	1 (0.9)	2 (1.8)	109
**Total**		**40 (0.4)**	**60 (0.6)**	**100 (1.0)**	**10,506**

* Canada: Total infection between physicians Chi square 27.8 df 3 p<0.0001; High vs. low certainty between physicians Fisher's exact test p=0.007

† New Zealand: Total infection between physicians Chi square 6.11 df 4 p=0.19: High vs. low certainty Fisher's between physicians’ exact test p=0.71

## RESULTS

We present the estimated risk of infection in the four vasectomy practices according to changes in clinical procedures and protocols that took place during the audit periods in Table-2. The overall risk of post-vasectomy infection was 0.8% in Canada, 2.1% of all patients and 2.8% of patients with routine one-week follow-up in Colombia, 1.0% in New Zealand, and 1.3% in United Kingdom.

In the Canadian practice, we did not observe clinically or statistically significant differences across audit periods, suggesting the limited effect of excising a short vas segment, applying routine topical antibiotic on the scrotal opening, and wearing surgical masks on the risk of infection.

At the Colombia practice, clinically and statistically significant differences were observed between the audit periods when ligation/excision combined with fascial interposition on the testicular segment (known as the Li occlusion technique) (11, 12) and when thermal mucosal cautery combined with facial interposition on the abdominal segment were performed for vas occlusion (13). When the latter technique was used, the risk of infection (0.9% of all patients and 1.2% of patients with routine one-week follow-up) was comparable to the risk observed in the other participating countries.

In New Zealand, there was limited difference in the risk of infection in the audit period when povidone iodine was used compared to the other audit periods. No clinically nor statistically significant difference was observed when comparing audit periods when sterile and non-sterile gloves were used.

The UK analysis revealed a statistically significant difference in infection risk across the 12 audit cycles, though the risk was low, ranging from 1.0% to 1.7%. There was no clinically significant trend across the 12 years. In the validation analysis performed on the 2012-2019 audit cycles, the infection risks of patients whose surgeons had a return rate of the four-month post-operative questionnaire of less than 10%, 10% to 33%, and 33% or more were 1.0%, 1.0%, and 2.0%, respectively. The risk of infection in the first (2016-2017) and the second (2017-2018) GP clinic audited were 0.7% and 1.2%, respectively.

Strata of infection risk observed between physicians and certainty of infections (low/high) were available for the practices in Canada and New Zealand (Table-3). In both practices, there were slight but similar variations in the total risk of infection between physicians. These differences were statistically significant in Canada but not in New Zealand. Overall, just over half of the infections were deemed to be of low certainty in both countries. In both sites, some physicians consistently reported higher proportions of low certainty infections. These differences between physicians reflecting certainty of infection were statistically significant only in Canada.

**Figure 1:**
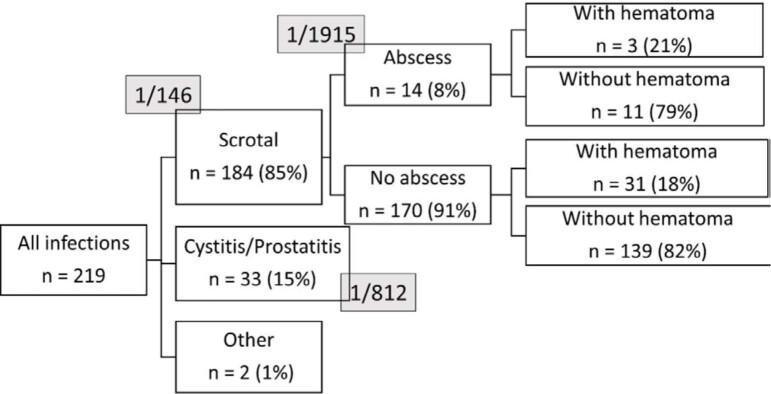
Types of post-vasectomy infection in Canada practice (n= 26,809 vasectomies).

Figure-1 shows the types of the 219 post-vasectomy infections encountered in Canada. Most (85%) were limited to the scrotal content but 15% were located in the prostate or urinary track. The risks of suffering any scrotal infection, a scrotal infection with an abscess, or a prostate or urinary track infection were 0.7%, 0.05%, and 0.1% respectively (Figure-1). Regardless of the presence of abscess, one in five scrotal infections were associated with a hematoma. Two other non-urinary or genital low probability infections were encountered in the electronic medical record; one was a fever of unknown origin and the other a heart valve infection occurring 14 and 28 days following the vasectomy, respectively.

## DISCUSSION

To our knowledge, this is the largest study on post-vasectomy infection ever reported. Analysis of our data demonstrates that about 1% of vasectomy patients suffered a post-vasectomy infection based on the proportion who received antibiotics for suspected or confirmed infection. A survey of US physicians published in 1982 – before the worldwide use of NSV – reported a 3.5% risk of infections among 65,155 vasectomies performed by 1,541 physicians who responded to the survey (14). However, our results are consistent with infection risk reported in the AUA practice guideline on vasectomy (1-2%). Our findings support the AUA recommendation that prophylactic antibiotics are not routinely indicated unless the patient presents a high risk of infection (3).

The estimated risk of infection was similar across practices in Canada, Colombia in recent years, New Zealand, and UK even though the settings, clinical procedures, and surgical techniques varied within and among each country. Interestingly, wearing a surgical mask did not seem to influence the risk of post-vasectomy infection. A Cochrane review has also concluded that there is no clear evidence that wearing disposable face masks modifies the risk of wound infections after clean surgery (15). Similarly, the use of sterile compared to non-sterile gloves did not appear to influence the risk of infection. This finding aligns with previously reported reviews of trials of non-sterile gloves versus sterile gloves in other minor surgical procedures (16–18).

The only clinically and statistically significant difference between factors that could influence the infection risk was observed in Colombia. The risk of infection decreased from an average of 2.6% of all patients /3.5% of patients with routine one-week follow-up in 2015-2018 to 0.9%/1.2% in 2019-2020 when surgeons changed their occlusion technique. They stopped using ligation and excision with FI on the testicular end after confirming in their practice the high risk of occlusion failure demonstrated in the Sokal et al. randomized trial (11, 12). They adopted the thermal cautery of the vas mucosa combined with FI on the prostatic end (13), recommended in clinical practice guidelines (3, 13, 19, 20). Colombian surgeons suggested that the use of three silk sutures with the former occlusion technique compared with only one silk suture with the currently used technique may contribute to this difference.

We were able to assess the type of infection in the Canadian practice. Although most infections were limited to scrotal content, a sizable proportion (15%) involved the prostate and urinary tract infection. Endogenous genital tract infection identified by pre-vasectomy semen culture have been associated with post-vasectomy infection (21). This could explain our findings in part, but the very low absolute risk (1/812) may not justify routine screening with pre-vasectomy semen culture. This study also highlighted the association of hematomas with scrotal infection with or without abscess. This finding supports the use of minimally invasive technique such as NSV to reduce both the risks of bleeding and infection as recommended in recent vasectomy guidelines (3, 19, 20, 22). We fortuitously identified a heart valve infection occurring in a patient who had a recent vasectomy. Infective endocarditis possibly caused by vasectomy has been previously reported (23–27).

Our study has limitations. Retrospective audit data were collected independently in the four practices. Information registered in medical records and ascertainment of cases at the time of diagnosis were not standardized. Certainty of post-vasectomy infection varies according to evaluation of symptoms and signs by the physician and follow-up method (telephone consultation or in-office visit). However, we agreed on a common definition of infection before data collection and analysis.

Our definition of infection - the use of antibiotics - may have led to an overestimation of post-vasectomy infection risk. Although we observed variations among physicians, globally, over half of vasectomized men from Canada and New Zealand who received antibiotics had low certainty of infection. The number of men with real post-vasectomy infections may then be lower than the number of all those receiving antibiotics. The risk could also have been underestimated. Men may have consulted general practitioners or emergency physicians and received antibiotics for non infectious post-vasectomy conditions. Such events may not be reported and go unnoticed by the vasectomy providers.

Our study has also many strengths. Our findings on infection risk were consistent across and within high volume practices of four countries. The sample size enhances precision in our estimates of risk. The involvement of multiple vasectomy providers of various level of experience using different procedures and vasectomy techniques indicates generalizability of our results. Importantly, our analysis demonstrates that a clear and consistent definition of post-vasectomy infection has yielded similar rates of post-vasectomy infection in various practices with varying techniques and procedures.

The risk of infection after vasectomy is low, about 1%, among international high-volume vasectomy practices performing the no-scalpel approach for vas isolation and various occlusion techniques recommended in vasectomy clinical practice guidelines (3, 19, 20, 22). Future research should continue to investigate variations in surgical and clinical procedures and associated risks of post-vasectomy infections.
